# The effects of culture on guideline discordant gestational weight gain: a systematic review protocol

**DOI:** 10.1186/s13643-015-0132-1

**Published:** 2015-11-03

**Authors:** Taru Manyanga, Danilo F. da Silva, Zachary M. Ferraro, Alysha L. J. Harvey, Shanna Wilson, Holly N. Ockenden, Kristi B. Adamo

**Affiliations:** Healthy Active Living and Obesity Research Group, Children’s Hospital of Eastern Ontario Research Institute, Ottawa, ON K1H 8L1 Canada; School of Human Kinetics, University of Ottawa, Faculty of Health Sciences, Ottawa, ON K1H 8L6 Canada; Department of Pediatrics, University of Ottawa, Faculty of Medicine, Ottawa, ON K1H 8L6 Canada; Department of Physical Education, State University of Maringa, Maringa, PR 87020-900 Brazil; Division of Maternal-Fetal Medicine, The Ottawa Hospital, General Campus, Ottawa, ON K1H 8L6 Canada; Faculty of Medicine, University of Ottawa, Ottawa, ON K1H 8L6 Canada

**Keywords:** Excessive, Inadequate, Gestational weight gain, Culture, IOM guidelines

## Abstract

**Background:**

A significant proportion of women exceeds or does not meet the Institute of Medicine’s gestational weight gain (GWG) guidelines. Inadequate, excessive GWG or weight loss during pregnancy is associated with an increased risk of negative maternal and fetal outcomes. Among the many determinants of GWG identified in the 2009 Institute of Medicine guidelines, culture was named as one of the few whose influence has not been fully explored. Some cultural beliefs may erroneously promote overeating as “eating for two” and discourage physical activity during pregnancy, but there is lack of empirical evidence on how culture affects GWG. The purpose of this systematic review is to examine the effects of culture on GWG.

**Methods/design:**

Ten electronic databases will be searched to identify studies reporting on the effects of culture on GWG. Grey literature, published conference abstracts, websites of relevant organizations and reference lists of included studies will also be searched. Studies that report on effects of culture, acculturation, ethnicity, race, nationality, ancestry and identity on GWG in adult women will be included. Quality of evidence will be evaluated using the grading of recommendations, assessment, development and evaluations (GRADE) approach to rating evidence. Study selection, data extraction and risk of bias assessment will be conducted by two independent reviewers, with disagreements being resolved by consensus or third party adjudication as needed. Formal meta-analyses will be conducted among included studies that are sufficiently statistically and clinically homogeneous.

**Discussion:**

This review will provide a comprehensive assessment and synthesis of current evidence and will draw attention to potential gaps where future research on the effects of culture on guideline discordant gestational weight gain remains to be conducted.

**Systematic review registration:**

PROSPERO CRD42015023399

## Background

As childhood obesity prevalence has risen to “epidemic” proportions in recent decades [1, 2], global public health efforts to curb this trend have intensified [3]. Childhood obesity is complex and multi-factorial [4, 5] which helps to explain the minimal to modest success of prevention and intervention strategies. Focusing on critical periods of growth and development such as pregnancy, a time when most women are likely to be more receptive to physical activity and dietary behaviour modification [5, 6], may be the key in tackling upstream causes of childhood obesity. Research has shown that a healthy pregnancy with adequate gestational weight gain (GWG) is important for the health of both mother and offspring [6, 7]. Inadequate or excessive gestational weight gain (hereinafter referred to as discordant GWG) has been associated with negative health outcomes for both the mother and child [8, 9]. Health risks associated with excessive GWG include but are not limited to the development of fetal overgrowth [10, 11], overweight/obesity in the child [12, 13], higher diastolic blood pressure at 4 years of age in the child [13], greater risk of hypertension [14] and gestational diabetes [15] in the mother. Inadequate GWG has been linked to pre-maturity and small for gestational age infants [16]. On the other hand, gestational weight loss even in pregnant women with obesity has been linked to higher odds of small for gestational age (SGA) infants [17].

To minimize risks associated with discordant GWG, the Institute of Medicine (IOM) provides specific recommendations for total and rate of GWG (Table 1), based on maternal pre-pregnancy body mass index (BMI) [9]. However, studies show that only 30 to 40 % of pregnant women meet these guidelines, with a significant proportion of women exceeding their recommended GWG [5, 6, 18]. Although not as prevalent as excessive gain, there is a subset of women who experience inadequate gain or weight loss during pregnancy, both of which are associated with pre-maturity and/or SGA neonates [17, 19, 20]. Predictors of GWG include pre-pregnancy weight [19], race/ethnicity [20], socioeconomic status (SES) and maternal health behaviour [21, 22], maternal age and parity [23]. A study of obesity trends in the USA [24] showed racial differences in the prevalence of obesity, lower in non-Hispanic White women compared to non-Hispanic Black women of childbearing (20–39 years) age. Headen et al. [25] demonstrated that Caucasian women had a decreased risk of inadequate GWG compared to normal weight and underweight Black and Hispanic women. On the other hand, Liu et al. [26] showed that Black women had 16–30 % lower odds of excessive GWG compared to White women, when analyses were run independent of weight status. Hispanic women presented even lower odds of excessive GWG than White women (29–46 %) without separating women according to their body mass index (BMI). In a study of Asian-American women, Cheng et al. [27] reported that overall, women of Asian descent had a higher risk of inadequate GWG compared to non-Hispanic White women. Among the different Asian subgroups (Indian, Chinese, Japanese, Filipino, Korean and Vietnamese), Japanese women had the highest (38.3 %) and Chinese women had the lowest (24.7 %) risk of inadequate GWG [26].

**Table 1:** IOM recommendations for total and rate of weight gain in pregnancy

Pre-pregnancy BMI	Recommended weight gain		Rates of weight gain per week in second and third trimester	
	Range in lb	Range in kg	Mean (range) in lb/week	Mean (range) in kg/week
Underweight <18.5 kg/m	28–40	12.5–18	1 (1–1.3)	0.51 (0.44–0.58)
Normal weight 18.5–24.9 kg/m	25–35	11.5–16	1 (0.8–1)	0.42 (0.35–.50)
Overweight 25.0–29.9 kg/m	15–25	7–11.5	0.6 (0.5–0.7)	0.28 (0.23–0.33)
Obese ≥30.0 kg/m	11–20	5–9	0.5 (0.4–0.6)	0.22 (0.17–0.27)

Over the last 15 years, numerous (>35) weight management interventions have been implemented during pregnancy with little impact on discordant GWG [5, 28, 29]. The continued presence of and in many cases increased prevalence of guideline discordant GWG may be related to the effects of factors such as culture whose influence on GWG is not well understood [9]. The revised 2009 IOM guidelines identified a number of determinants of GWG including maternal, psychological, familial, cultural, health services and environmental factors [9]. Although culture was identified as an important determinant of GWG, the IOM acknowledged the absence of empirical evidence based on pre-pregnancy BMI on the magnitude of its influence to draw any conclusions [9]. This may be partly because culture is notoriously difficult to describe [30], with no consensus definition among experts [31]. Furthermore, globalization and migration over time have led to mixing of cultures and acculturation (adoption of values and customs of other groups due to immigration), further confounding cultural distinctions [32]. Although there is no consensus among experts, culture, as defined by Spencer-Oatey [33], is “a fuzzy set of basic assumptions and values, orientations to life, beliefs, policies, procedures and behavioural conventions that are shared by a group of people, and that influence (but do not determine) each member’s behaviour and his/her interpretations of the ‘meaning’ of other people’s behaviour”. In a subsequent study, Spencer-Oatey [30] accurately noted the various and lack of consensus definitions of culture and contends that race, nationality, ethnicity or ancestry are not necessarily synonymous with culture. The lack of a consensus definition however, has led researchers examining the effects of culture on GWG to use these descriptors of identity as its proxies. As such, this systematic review will use race, ethnicity, ancestry, acculturation and nationality interchangeably with culture. To the best of our knowledge, no systematic review has been published on the effects of culture on GWG. GWG is defined as weight before delivery (as stated by each included study) minus the weight measured at booking or self-reported pre-pregnancy weight. Therefore, the purpose of this systematic review is to synthesize and analyze data from randomized controlled trials and observational and cohort studies on the effects of culture on GWG.

## Methods/Design

### Structured research question

What is the effect of culture on guideline discordant (inadequate or excessive) gestational weight gain or gestational weight loss?

### PICOS

#### Population

- The majority (≥80 %) of participants are adult (18–49 years) pregnant women.

#### Intervention/exposure

- Ethnicity/culture/ancestry/nationality/race

#### Comparators

- Two or more culturally distinct groups

### Outcomes

The outcome of interest in this review is inadequate, adequate or excessive GWG. Studies which do not assess or report GWG will be excluded.

#### Primary outcome

- Excessive gestational weight gain (more than recommendations in Table 1)
- Inadequate gestational weight gain (less than recommendations in Table 1)

#### Secondary outcomes

- Gestational weight loss (GWL)
- Medical complications of pregnancy (e.g. gestational diabetes mellitus, pre-eclampsia, and pregnancy-induced hypertension)
- Maternal outcomes (e.g. mode of delivery, induction of labour, infection, length of stay in hospital, postpartum haemorrhage, and anaesthesia complications
- Fetal outcomes (e.g. macrosomia, LGA, shoulder dystocia, SGA, pre-maturity)

##### Study design

A systematic review of published and unpublished randomized controlled trials, observational and cohort studies will be undertaken to examine the effects of culture on GWG. In this review, we will adhere to the reporting guidelines of the Preferred Reporting Items for Systematic Reviews and Meta-Analyses (PRISMA) statement [34].

### Study registration

This systematic review has been prospectively registered with PROSPERO (registration number: CRD42015023399; http://www.crd.york.ac.uk/PROSPERO/register_new_review.asp?RecordID=23399&UserID=3733).

### Eligibility criteria

#### Inclusion criteria

1. Studies in which participants were pregnant women (≥18 years)
2. Singleton pregnancy
3. Prospective randomized controlled trials, observational and cohort studies.
4. Reported (self-reported or directly measured) gestational weight gain.

#### Exclusion criteria

1. Studies involving animals.
2. Studies in which participants’ ethnicity/culture/ancestry/nationality/race was not reported.
3. Studies in which gestational weight was not reported.
4. Multiparous pregnancy.

### Search methods for identifying studies

A comprehensive search strategy was developed in consultation with a Health Science Librarian (EW). We will perform searches of the following electronic databases: Ovid MEDLINE (R) In-Process & Other Non-Indexed Citations and Ovid MEDLINE (R) 1946 to present, Embase (Ovid), Cochrane Central Register of Controlled Trials (present), CINAHL (EbscoHost), PyscINFO and Sociological Abstracts. In addition, we will also search the Literature Latino-Americana e do Caribe em Ciencias da Saude (LILACS), IBECS, Cuba Medicina (CUMED), which are traditionally non-English databases. The search terms for Ovid MEDLINE are presented in Table 2 and will be modified as needed to suit the indexing systems of other databases. Explosion and Boolean terms will be used to expand searches. Forward searches will be performed in Scopus and Web of Science to identify additional relevant citations. Published abstracts from conference (Developmental Origins of Health and Disease (DOHaD), Global Maternal Newborn Health Conference) proceedings will be searched. Grey literature (CADTH’s Grey matters) will also be searched and included if it meets the inclusion criteria. Lastly, reference lists of relevant systematic reviews and included trials will be hand-searched for possible relevant citations. We will not impose language restrictions but accrue the relevant translation as required. Non-English studies that are beyond our translation or cost capacity will be compiled in an appendix but will not be incorporated in any of the analyses.

**Table 2:** Sample Ovid® Medline search strategy

1	Pregnancy/
2	Pregnant women/
3	Pregnancy outcomes/ or pregnancy complications/
4	(Pregnant* or gestation*).ti,ab.
5	or/1–4
6	Exp body weight changes
7	(Weight adj2 chang*).ti,ab.
8	(Weight adj2 (gain* or increase*)).ti,ab.
9	(Weight adj2 (loss or lose or losing or decrease*)).ti,ab.
10	((BMI or body mass index) adj2 (increase* or decrease* or change* or loss*)).ti,ab.
11	or/6–10
12	5 and 11
13	Culture/
14	Acculturation/
15	Cross-cultural comparison/
16	Cultural characteristics/
17	Cultural diversity/
18	Cultural evolution/
19	Exp ethnic groups/
20	Exp continental population groups/
21	(Acculturation* or sociocultural* or socio-culture* or cultural* or ethnic* or ancestry* or ethnoracial*).ti,ab.
22	Or/13–21
23	12 and 22

*different variations/forms of the term

### Study selection

Articles retrieved from the search will be imported into EndNote (X6) [35] where duplicates will be removed using the “duplicate” function. After removal of duplicates, studies will be exported to *Covidence* [36] for screening, selection and data extraction. A two-step process will be used for study selection. First, two reviewers (TM and DFdS) will independently screen the titles and abstracts (when available) of search results to determine if a study meets the general inclusion criteria. Each report will be classified as include, exclude or unsure. Discrepancies between the two reviewers will be resolved through consensus dialogue or third party adjudication by the content expert as needed. The full texts of all reports classified as “include” or “unsure” will be retrieved for formal review. Next, the two reviewers will independently assess the full text of each study by using a standardized form that outlines the pre-determined inclusion and exclusion criteria. The form will be pilot tested on a sample of studies. After the form is tested, disagreements will be resolved by a discussion between the two reviewers or by third party adjudication, as needed. A PRISMA flow diagram that illustrates the records under consideration and those selected or excluded will be constructed over the course of the review and justification for excluding studies will be provided.

### Data extraction

Data will be extracted by using a standardized form. The form will be created with input from the reviewer team members and pilot tested on a sample of studies. The data extraction form will be modified based on the pilot-test feedback to ensure that complete information is obtained. Data from full-text study reports will be extracted by two reviewers (TM and DFdS) independently with conflicts resolved through consensus, and with the assistance of a third party if consensus cannot be achieved. The following data will be extracted from each study: author identification, year of publication, language of publication, source of study funding, study design, region and country where study was conducted, setting, methodological quality criteria (see below), study population (including study inclusion and exclusion criteria), number of participants, participant characteristics (age, pre-pregnancy weight), exposure details (ethnicity/cultural background), relevant covariates and results reported for the outcome (GWG) of interest.

### Contacting authors for missing data

We will contact all corresponding authors of included studies with incomplete, unclear or missing data and request them to provide further written description of such content.

### Assessing quality of evidence

Two independent assessors (TM and DFdS) will conduct quality of evidence assessment with disagreements resolved by consensus or third party adjudication when needed. Quality of evidence will be assessed using the grading of recommendations, assessment, development and evaluation (*GRADE*) *approach* [37] to rating evidence. GRADE specifies four categories: high, moderate, low and very low which are applied to a body of evidence, not to individual studies. The approach addresses five reasons (risk of bias, inconsistency, indirectness, imprecision, and publication bias) to possibly rate down the quality of evidence and three (large effect, dose response and all plausible residual confounding accounted for) to possibly rate up the quality [37]. Randomized controlled trials will begin as high quality while observational studies begin as low quality. Studies will be rated up or down depending on how they score on the set GRADE criteria. In addition, information on the source of funding will be collected for each study. Information regarding methodological quality (e.g. low risk vs. unclear/high risk of bias, RCT vs. other studies, and published vs. unpublished/grey literature) will be used to guide sensitivity analyses and explore sources of heterogeneity.

### Anticipated analyses

Data from included studies will be analyzed using RevMan (version 5.3) [38]. Statistical heterogeneity of the data will be explored and quantified using the I-squared tests. If data are highly heterogeneous (*I*^2^ ≥ 75 %), they will not be pooled, and a formal meta-analysis will not be conducted. with values ≥75 % used to indicate high heterogeneity [39]. If significant heterogeneity is suspected, further exploration including viewing overlapping confidence intervals and subgroup analysis will be conducted. A formal meta-analysis will be conducted if the data are found to be statistically and clinically homogenous. Publication bias will be assessed using the funnel plot technique. Effects of ethnicity/culture on GWG (calculated as the difference between pre-pregnancy weight and post-delivery weight) will be evaluated. Mean difference in excessive or inadequate GWG (kg) between groups will be used as the effect size for analyses. Pooled continuous data will be expressed as a weighted mean difference (WMD), with corresponding 95 % confidence intervals (CI). Pooled dichotomous data (number or percentage of women with discordant GWG) will be presented as an odds ratio (OR) with corresponding 95 % CI.

### Subgroup/sensitivity analysis

In addition to the primary analyses, the following a priori subgroup and sensitivity analyses are proposed for primary outcomes. Such analyses will depend on the number of studies included and the availability of appropriate outcomes and covariates.

### Methodological

- Quality (low risk of bias vs. unclear/ high risk of bias)
- Source (published vs. grey literature/ abstracts)
- Study type (randomized controlled trial vs. other studies)
- Outcome measures (studies with self-reported vs. directly measured GWG)
- Centres (single vs. multiple centres)
- No. of participants (≤100 vs. >100)

### Clinical

- Year of publication (before 2009 vs. 2009 onwards)
- Pre-pregnancy weight (normal vs. overweight; normal vs. obese; normal vs. underweight)
- Populations (healthy vs. women with pregnant-related complications)
- Socioeconomic status (low vs. medium SES; low vs. high SES)
- Region (Latin America; Africa; Asia vs. North America/Europe; developing vs. developed countries)
- Acculturation (0–5 vs. >5 years)
- Setting (urban vs. rural)

## Discussion

To the best of our knowledge, this will be the first systematic review to critically examine and synthesize the available literature assessing the relationship between culture/ethnicity and GWG. This review will provide a comprehensive assessment and synthesis of current evidence to date and will draw attention to potential gaps where future research on the effects of culture on guideline discordant GWG remains to be conducted. We anticipate that this systematic review will provide useful information on the effects of culture on GWG for a variety of stakeholders including clinicians, researchers and policy makers. Given that the revised IOM guidelines [9] identified culture as one of the key determinants of GWG whose influences were not well understood thus needed to be studied, this review may help in that regard.

## Abbreviations

BMI: body mass index
GWG: gestational weight gain
GWL: gestational weight loss
IOM: Institute of Medicine
LGA: large for gestational age
PICOS: populations interventions comparators outcomes study design
PRISMA: preferred reporting items for systematic reviews and meta-analyses
SGA: small for gestational age
SES: socioeconomic status
WMD: weighted mean difference
